# Strategies to Improve Health Care Provider Prescription of and Patient Adherence to Guideline-Recommended Cardiovascular Medications for Atherosclerotic Occlusive Disease: Protocol for Two Systematic Reviews and Meta-Analyses of Randomized Controlled Trials

**DOI:** 10.2196/60326

**Published:** 2025-01-16

**Authors:** Aidan M Kirkham, Dean A Fergusson, Justin Presseau, Daniel I McIsaac, Risa Shorr, Derek J Roberts

**Affiliations:** 1 Division of Vascular and Endovascular Surgery Department of Surgery University of Ottawa Ottawa, ON Canada; 2 Clinical Epidemiology Program The Ottawa Hospital Research Institute The Ottawa Hospital Ottawa, ON Canada; 3 School of Epidemiology & Public Health Faculty of Medicine University of Ottawa Ottawa, ON Canada; 4 Department of Anesthesiology and Pain Medicine University of Ottawa Ottawa, ON Canada; 5 Institute for Clinical Evaluative Sciences Toronto, ON Canada; 6 Learning Services The Ottawa Hospital Ottawa, ON Canada

**Keywords:** coronary artery disease, cerebrovascular disease, peripheral artery disease, polyvascular disease, underprescription, nonadherence, implementation strategy, adherence-supporting strategy, statins, antiplatelets, antihypertensives, guideline-recommended medications, implementation, atherosclerosis, patient adherence, RCT, randomized controlled trials, PRISMA

## Abstract

**Background:**

In patients with atherosclerotic occlusive diseases, systematic reviews and meta-analyses of randomized controlled trials (RCTs) report that antiplatelets, statins, and antihypertensives reduce the risk of major adverse cardiac events, need for revascularization procedures, mortality, and health care resource use. However, evidence suggests that these patients are not prescribed these medications adequately or do not adhere to them once prescribed.

**Objective:**

We aim to systematically review and meta-analyze RCTs examining the effectiveness of implementation or adherence-supporting strategies for improving health care provider prescription of, or patient adherence to, guideline-recommended cardiovascular medications in patients with atherosclerotic occlusive disease.

**Methods:**

We designed and reported the protocol according to the PRISMA-P (Preferred Reporting Items for Systematic Review and Meta-Analysis-Protocols) statement. We will search MEDLINE, Embase, The Cochrane Central Register of Controlled Trials, PsycINFO, and CINAHL from their inception. RCTs examining implementation or adherence-supporting strategies for improving prescription of, or adherence to, guideline-recommended cardiovascular medications in adults with cerebrovascular disease, coronary artery disease, peripheral artery disease, or polyvascular disease (>1 of these diseases) will be included. Two investigators will independently review identified titles/abstracts and full-text studies, extract data, assess the risk of bias (using the Cochrane tool), and classify implementation or adherence-supporting strategies using the refined Cochrane Effective Practice and Organization of Care (EPOC) taxonomy (for strategies aimed at improving prescription) and Behavior Change Wheel (BCW; for adherence-supporting strategies). We will narratively synthesize data describing which implementation or adherence-supporting strategies have been evaluated across RCTs, and their reported effectiveness at improving prescription of, or adherence to, guideline-recommended cardiovascular medications (primary outcomes) and patient-important outcomes and health care resource use (secondary outcomes) within refined EPOC taxonomy levels and BCW interventions and policies. Where limited clinical heterogeneity exists between RCTs, estimates describing the effectiveness of implementation or adherence-supporting strategies within different refined EPOC taxonomy levels and BCW interventions and policies will be pooled using random-effects models. Stratified meta-analyses and meta-regressions will assess if strategy effectiveness varies by recruited patient populations, prescriber types, clinical practice settings, and study design characteristics. GRADE (Grading of Recommendations, Assessment, Development, and Evaluation) will be used to communicate evidence certainty.

**Results:**

The search was completed on June 6, 2023. Database searches and the PubMed “related articles” feature identified 4319 unique citations for title/abstract screening. We are currently screening titles/abstracts.

**Conclusions:**

These studies will identify which implementation and adherence-supporting strategies are being used (and in which combinations) across RCTs for improving the prescription of, or adherence to, guideline-recommended cardiovascular medications in adults with atherosclerotic occlusive diseases. They will also determine the effectiveness of currently trialed implementation and adherence-supporting strategies, and whether effectiveness varies by patient, prescriber, or clinical practice setting traits.

**Trial Registration:**

PROSPERO CRD42023461317; https://www.crd.york.ac.uk/prospero/display_record.php?RecordID=461317; PROSPERO CRD42023461299; https://www.crd.york.ac.uk/prospero/display_record.php?RecordID=461299

## Introduction

### Background

Atherosclerotic occlusive disease is common worldwide, increasing in global prevalence, and characterized by arterial stenosis (narrowing) and occlusion secondary to the buildup of lipid-rich plaque within arterial walls [1]. This disease may be further categorized by anatomic location, with atherosclerotic plaque buildup within the arteries of the extracranial and intracranial circulation, heart, and peripheral arteries being referred to as cerebrovascular disease (CVD), coronary artery disease (CAD), and peripheral artery disease (PAD), respectively [1]. An estimated 101 million [2], 197 million [3], and 113 million [4] individuals have CVD, CAD, and PAD worldwide, respectively. CVD, CAD, and PAD are associated with a significantly increased risk of hospitalization; need for coronary, carotid, or lower limb revascularization; major adverse cardiac events (MACEs) and major adverse limb events (MALEs); stroke; and cardiovascular and all-cause mortality [5,6].

International, evidence-based clinical practice guidelines strongly and consistently recommend that adults with CVD, CAD, PAD, and polyvascular disease be prescribed and take antiplatelets and statins (ie, hydroxymethylglutaryl-CoA reductase inhibitors) [7-11]. They also recommend antihypertensives for those patients with concurrent hypertension [7-11]. These recommendations are based on high-quality data from randomized controlled trials (RCTs) and systematic reviews and meta-analyses of RCTs that reported a significantly lower risk of MACE; MALE; need for coronary, carotid, and lower limb revascularization procedures; cardiovascular and all-cause mortality; and health care resource use when these patients are prescribed these medications [12-14].

Despite this, evidence suggests that many patients with atherosclerotic occlusive disease are not prescribed these medications adequately or do not adhere to them after prescription [15-18]. More specifically, only approximately 40% to 90% of patients with any form of atherosclerotic occlusive disease are prescribed any antiplatelet, 30% to 60% any statin, 40% to 60% any antihypertensive, and 10% to 40% all three guideline-recommended cardiovascular medications [15,16]. Studies also report that patient adherence percentages to these medications range from ~70% to 80% for antiplatelets, ~50% to 60% for statins, ~40% to 70% for antihypertensives, and ~20% to 40% for all three guideline-recommended cardiovascular medications [17,18]. Those with PAD are also significantly less likely to be prescribed these medications and adhere to them compared to those with CVD and CAD [19]. The increased frequency at which patients with PAD are underprescribed and nonadherent to guideline-recommended cardiovascular medications may partially explain why studies demonstrate persistently higher risks of cardiovascular-related hospitalization (eg, for atherothrombotic events), MACE, stroke, and cardiovascular mortality in patients with PAD compared to those with CVD or CAD [20].

To improve the use of guideline-recommended cardiovascular medications among patients with atherosclerotic occlusive disease, RCTs [21-48] have evaluated implementation strategies (interventions directed at the health system and health care providers) and adherence-supporting strategies (interventions aimed at patients directly) [49] to improve health care provider prescription of and patient adherence to guideline-recommended cardiovascular medications, respectively. However, the design [21-24], length of follow-up [24,27,31], setting [21,24-26], and implementation or adherence-supporting strategies examined [21,26,27,30-32,36,40-48] varies considerably across published trials. Existing RCTs have also examined implementation and adherence-supporting strategies aimed at improving prescription of or adherence to different medication classes (antiplatelets [25,28,29], statins [27-29], antihypertensives [25,28,42], or all guideline-recommended medications [27,29,43,44]) among patients with different diagnoses (CVD [31-33], CAD [24,26,27], PAD [34,35], and polyvascular disease [36,37]). Further, the implementation and adherence-supporting strategies examined within published trials target different types of health care providers (ie, cardiovascular specialists [38,39] or noncardiovascular specialists [21,23]) and health care provider behaviors [31,33,38,40]. Given this interstudy heterogeneity, evidence-synthesis efforts are needed to interpret the results of the above RCTs collectively.

### Rationale

Two updated, comprehensive, and methodologically rigorous systematic reviews and meta-analyses are needed to determine the effectiveness of implementation and adherence-supporting strategies for improving health care provider prescription of, and patient adherence to, guideline-recommended cardiovascular medications for atherosclerotic occlusive diseases. All forms of atherosclerotic occlusive disease (CVD, CAD, PAD, and polyvascular disease) will be examined within both systematic reviews as the underlying pathophysiology [50-52], and guideline-recommended cardiovascular medications for medical management of these diseases [7-11] are nearly identical. Further, patients with these atherosclerotic occlusive diseases tend to have similar demographics (ie, frequently past or current smokers, often male, and typically over 65 years of age) and comorbidities [53-55]. Finally, these diseases often coexist together [56,57], and similar prescribers are involved in their medical management [58-60]. The results of these systematic reviews will help identify promising implementation and adherence-supporting strategies for each and all classes of cardiovascular medications recommended for patients with atherosclerotic occlusive diseases. The systematic reviews will facilitate an effective comparison of implementation and adherence-supporting strategies by classifying them according to established taxonomies [61,62].

### Objectives

The primary objectives of the two systematic reviews are to synthesize the effectiveness of implementation and adherence-supporting strategies for improving health care provider prescription of, and patient adherence to, guideline-recommended cardiovascular medications among patients with atherosclerotic occlusive disease, including CVD, CAD, PAD, or polyvascular disease. We conceptualized the desired behavior changes for our primary objectives and key actors involved in bringing about these desired behavior changes using the Action, Actor, Context, Target, Time (AACTT) framework [63] (see Table 1 for the underprescription systematic review AACTT framework and Table 2 for the nonadherence systematic review AACTT framework).

**Table 1:** AACTT^a^ behavior specification framework [63] for the systematic review of implementation strategies for improving health care provider prescription of guideline-recommended cardiovascular medications to adults with atherosclerotic occlusive disease.

AACTT domains	Examples among the systematic review of implementation strategies for improving health care provider prescription of cardiovascular medications
Action	Prescription of guideline-recommended cardiovascular medications (antiplatelets, statins, or antihypertensives)
Actor	Health care providers responsible for managing patients with atherosclerotic occlusive disease
Context	Any setting where prescribers are responsible for prescribing medication to patients with atherosclerotic occlusive diseases (eg, primary care practices, tertiary academic hospitals, or pharmacies)
Target	Patients with an atherosclerotic occlusive disease (cerebrovascular disease, coronary artery disease, peripheral artery disease, or polyvascular disease) in whom one or more guideline-recommended cardiovascular medications is indicated
Time	During encounters between prescribers and patients with atherosclerotic occlusive disease

^a^AACTT: Action, Actor, Context, Target, Time.

**Table 2:** AACTT^a^ behavior specification framework [63] for the systematic review of adherence-supporting strategies for improving patient adherence to guideline-recommended cardiovascular medications in adults with atherosclerotic occlusive disease.

AACTT domains	Examples among the systematic review of adherence-supporting strategies for improving patient adherence to cardiovascular medications
Action	Adherence to guideline-recommended cardiovascular medications for atherosclerotic occlusive disease (antiplatelets, statins, or antihypertensives)
Actor	Patients with a diagnosed atherosclerotic occlusive disease (cerebrovascular disease, coronary artery disease, peripheral artery disease, or polyvascular disease)
Context	Any setting in which patients (or their caregivers) are responsible for managing their own medications
Target	Patients who have been prescribed one or more guideline-recommended cardiovascular medications for management of their atherosclerotic occlusive disease by a health care professional
Time	Following prescription of guideline-recommended cardiovascular medications by a health care professional

^a^AACTT: Action, Actor, Context, Target, Time.

The AACTT framework [63] was developed to describe the action (ie, the behavior to change), actor (ie, who is involved in performing the behavior), context (ie, the physical, social, or emotional settings where the behavior is performed), target (ie, the person or people for or with whom the behavior is performed), and time (ie, when the behavior is performed) of behaviors targeted for change in clinical practice. Describing desired behavior changes using the five elements of the AACTT framework [63] provides a concise overview of which individuals at what organizational levels need to do what differently to ensure the effective implementation of desired best practices. Secondary objectives are to determine whether (1) the effectiveness of implementation or adherence-supporting strategies at improving health care provider prescription of or increasing patient adherence to guideline-recommended cardiovascular medications for atherosclerotic occlusive disease varies by patient characteristics, prescriber traits, medication classes, atherosclerotic occlusive disease diagnoses, or clinical settings; and (2) improved health care provider prescription of or patient adherence to guideline-recommended cardiovascular medications improves patient outcomes and reduces health care resource use.

## Methods

### Registration and Reporting

This protocol was created following recommendations from the PRISMA-P (Preferred Reporting Items for Systematic Reviews and Meta-Analyses Protocols) statement [64] (a completed PRISMA-P checklist is provided in Multimedia Appendix 1). The protocols for the systematic reviews of implementation and adherence-supporting strategies for improving health care provider prescription and patient adherence were registered separately on PROSPERO (underprescription systematic review: CRD42023461317; nonadherence systematic review: CRD42023461299). We chose to create a single protocol paper describing the methods of the two planned systematic reviews and meta-analyses as we felt that their background, rationale, and methods overlap significantly. However, as scout searches suggested that one systematic review may include over 100 RCTs, we plan to report their results separately to summarize the results of these evidence syntheses in sufficient detail.

### Eligibility Criteria

Eligibility criteria for the two systematic reviews were created according to the Population, Intervention, Comparison, Outcome, and Design framework [65]. Their specific inclusion and exclusion criteria are presented in Table 3 (underprescription systematic review) and Table 4 (nonadherence systematic review).

**Table 3:** Inclusion and exclusion criteria for systematic review examining implementation strategies for improving health care provider prescription of guideline-recommended cardiovascular medications to patients with atherosclerotic occlusive disease.

PICOD^a^ framework domain	Inclusion criteria^b^	Exclusion criteria^c^
Population	Prescribers (ie, physicians, nurse practitioners, or pharmacists) involved in the management of adults (≥18 years of age) with atherosclerotic occlusive disease (defined as cerebrovascular disease, coronary artery disease, peripheral artery disease, or polyvascular disease).	>20% of the patient population had nonatherosclerotic cardiovascular disease (eg, aneurysmal disease). This 20% threshold was selected based on prior literature [66]. Studies examining the prescription of nonguideline-recommended medications. Prescribers involved in the management of people exclusively <18 years of age.
Intervention	Any implementation strategy aimed at increasing health care provider prescription of guideline-recommended cardiovascular medications to patients with atherosclerotic occlusive disease. An implementation strategy will be defined as any “method or technique to enhance the adoption, implementation, and sustainability of a clinical program or practice” [49].	Studies examining implementation strategies directed exclusively at patients.
Comparison	Health care providers prescribing guideline-recommended cardiovascular medications to patients with atherosclerotic occlusive disease without the use of an implementation strategy, patients receiving the same implementation strategy at a lower intensity or shorter duration than the intervention group, or patients receiving a different implementation strategy than the intervention group.	Studies examining implementation strategies directed exclusively at patients.
Outcomes	Primary^d^: Effect estimates (or data required to calculate) describing differences in health care provider prescription between implementation strategy and control arms of each (ie, antiplatelets, statins, or antihypertensives) or all classes of guideline-recommended cardiovascular medications. Secondary^d^: Effect estimates (or data required to calculate these) describing differences between patients in the implementation strategy and control arms regarding adverse clinical outcomes (eg, major adverse cardiac events, revascularization procedures, amputations, cardiovascular mortality, or all-cause mortality) and health care resource use outcomes (eg, emergency department visits, hospital admission or readmission, or length of hospitalization).	Studies not reporting effect estimates (or data required to calculate) describing differences in guideline-recommended cardiovascular medication prescription between prescribers in the implementation strategy and control arms.
Design	Study designs will include RCTs^e^ and cluster-RCTs.	Nonrandomized studies (eg, prospective cohort studies). Studies published in abstract form only and unpublished studies (ie, gray literature).

^a^PICOD: Population, Intervention, Comparison, Outcome, and Design.

^b^Inclusion criteria formulated according to the Population, Intervention, Comparison, Outcome, and Design framework for posing clinical questions [65].

^c^Studies meeting one or more of the following criteria will be excluded.

^d^Effect estimates (and measures of variation, such as 95% CIs) will be extracted as reported and may include weighted mean differences or standardized mean differences for continuous outcomes; odds ratios, relative risks, and risk differences for binary outcomes; and hazard ratios for time-to-event data.

^e^RCT: randomized controlled trial.

**Table 4:** Inclusion and exclusion criteria for systematic review examining adherence-supporting strategies for improving patient adherence to guideline-recommended cardiovascular medications in patients with atherosclerotic occlusive disease.

PICOD^a^ framework domain	Inclusion criteria^b^	Exclusion criteria^c^
Population	Adults (≥18 years of age) with atherosclerotic occlusive disease (cerebrovascular disease, coronary artery disease, peripheral artery disease, or polyvascular disease) prescribed one or more guideline-recommended cardiovascular medications (eg, statins, antihypertensives, or antiplatelets).	>20% of patients had nonatherosclerotic cardiovascular disease (eg, aneurysmal disease). This threshold was selected based on prior literature [66]. Studies where ≥20% of patients were pediatrics (<18 years of age). Studies examining adherence to medications not recommended within relevant clinical practice guidelines.
Intervention	Any adherence-supporting strategy aimed at increasing adherence to guideline-recommended cardiovascular medication in patients with atherosclerotic occlusive disease. An adherence-supporting strategy will be defined as any “method or technique to enhance the adoption, implementation, and sustainability of a clinical program or practice” [49].	Studies examining adherence-supporting strategies directed at health care providers or the health care system without components directed toward patients.
Comparison	Patients taking guideline-recommended cardiovascular medications for atherosclerotic occlusive disease management without a concurrent adherence-supporting strategy, patients receiving a different adherence-supporting strategy than the intervention group, or patients receiving the same adherence-supporting strategy at a lower intensity or shorter duration than the intervention group.	Studies examining adherence-supporting strategies directed at health care providers or the health care system without components directed toward patients.
Outcomes	Primary^d^: Effect estimates (or data required to calculate) describing differences in medication adherence between adherence-supporting strategy and control arms of each (eg, antiplatelets, statins, or antihypertensives) or all classes of guideline-recommended cardiovascular medications. Secondary^d^: Effect estimates (or data required to calculate these) describing differences between patients in the adherence-supporting strategy and control arms regarding adverse clinical outcomes (eg, major adverse cardiac events, revascularization procedures, amputations, cardiovascular mortality, or all-cause mortality) and health care resource use outcomes (eg, emergency department visits, hospital admission or readmission, or length of hospitalization).	Studies not reporting effect estimates (or data required to calculate) describing differences in medication adherence between patients in the intervention and control arms.
Design	RCTs^e^ and cluster-RCTs.	Nonrandomized studies (eg, case-control studies). Unpublished studies (ie, gray literature) and studies published in abstract form only.

^a^PICOD: Population, Intervention, Comparison, Outcome, and Design.

^b^Inclusion criteria formulated according to the Population, Intervention, Comparison, Outcome, and Design framework for posing clinical questions [65].

^c^Studies meeting one or more of the following criteria will be excluded.

^d^Effect estimates (and measures of variation, such as 95% CIs) will be extracted as reported and may include weighted mean differences or standardized mean differences for continuous outcomes; odds ratios, relative risks, and risk differences for binary outcomes; and hazard ratios for time-to-event data.

^e^RCT: randomized controlled trial.

Guideline-recommended cardiovascular medications will be defined as any medications recommended within evidence-based clinical practice guidelines cited within the included studies. In studies where evidence-based clinical practice guidelines are not cited, guideline-recommended cardiovascular medications will be defined as antiplatelets (eg, aspirin or clopidogrel), statins, and antihypertensives (eg, angiotensin-converting enzyme inhibitors, angiotensin receptor blockers, β-blockers, calcium-channel blockers, or thiazide diuretics), as these medications are consistently recommended for individuals with CVD, CAD, PAD, and polyvascular disease across multiple evidence-based clinical practice guidelines [7-11].

### Clinical Questions

#### Primary Clinical Questions

The primary clinical questions are as follows:

1. What is the effectiveness of different implementation strategies for improving health care provider prescription of guideline-recommended cardiovascular medications to adults (≥18 years of age) with atherosclerotic occlusive disease, including CVD, CAD, PAD, or polyvascular disease?
2. What is the effectiveness of adherence-supporting strategies for increasing adherence to guideline-recommended cardiovascular medications among adults with atherosclerotic occlusive disease?

#### Secondary Clinical Questions

The secondary clinical questions are as follows:

1. Does the effectiveness of implementation and adherence-supporting strategies for improving health care provider prescription of and patient adherence to guideline-recommended cardiovascular medications to adults with atherosclerotic occlusive diseases vary by study design, prescriber characteristics, patient characteristics, atherosclerotic occlusive disease diagnosis, or clinical practice setting?
2. Does the use of implementation or adherence-supporting strategies aimed at improving health care provider prescription of or patient adherence to guideline-recommended cardiovascular medications to adults with atherosclerotic occlusive disease improve patient outcomes or reduce health care resource use?

### Information Sources

We will search MEDLINE, Embase, The Cochrane Central Register of Controlled Trials, PsycINFO, and CINAHL from their inception. We will also use the PubMed “related articles” feature and manually search reference lists of included studies and relevant reviews identified during the search.

### Search Strategy

Our electronic search strategy was designed in consultation with an information scientist. Using combinations of Medical Subject Headings (National Library of Medicine) or Emtree (Elsevier) terms and keywords, we constructed search filters covering the themes “atherosclerotic occlusive disease” and “medication prescription or adherence.” These search filters were then combined with a validated RCT search filter from the Cochrane Handbook for Systematic Reviews of Interventions [67]. The search strategy was subsequently piloted and refined by adding additional thesaurus or indexing terms to the nonsearch filter themes when new, relevant citations were located during iterative pilot searches. The penultimate search strategies were peer-reviewed by another medical librarian in accordance with the Peer Review of Electronic Search Strategies framework [68] (see Multimedia Appendix 2 for final search strategies).

### Study Selection and Management

Titles and abstracts of identified citations will be reviewed by two independent reviewers using Rayyan Systematic Review Software [69]. Citations deemed potentially relevant by either reviewer during title/abstract screening will be subject to independent full-text review using relevant inclusion and exclusion criteria (see Tables 3 and 4). Disagreements regarding study inclusion will be resolved by consensus.

### Outcomes

The primary outcome for the underprescription systematic review will be the health care provider’s prescription of each (eg, antiplatelets, statins, or antihypertensives) or all classes of guideline-recommended cardiovascular medications between the implementation strategy and control arms. The primary outcome of the nonadherence systematic review will be the patient adherence to each (eg, antiplatelets, statins, or antihypertensives) or all classes of guideline-recommended cardiovascular medications between the adherence-supporting strategy and control arms. Secondary outcomes for both systematic reviews will be differences in patient outcomes and health care resource use between the implementation or adherence-supporting strategy and control arms. Patient outcomes will include MACE [70] (defined as nonfatal myocardial infarction, stroke, or cardiovascular mortality), MALE [70] (defined as limb ischemia requiring revascularization or above-ankle lower limb amputation), stroke, revascularization procedures, amputations, patient-reported outcome measures, cardiovascular mortality, all-cause mortality, and any other patient-important outcomes reported by study authors. Health care resource use outcomes will include emergency department visits, hospital admission or readmission, length of hospitalization, and any other health care resource use outcomes reported by study authors.

### Data Items and Selection Process

Two investigators will independently extract data using a predesigned electronic data extraction spreadsheet (see Tables 5 and 6 for data items to be extracted from the underprescription and nonadherence systematic reviews, respectively).

When potentially relevant articles published in languages other than English are identified, translators will be recruited to review the full texts and extract data if identified studies meet inclusion criteria. Effect estimates and their 95% CIs (or data required to calculate these measures) describing associations between implementation or adherence-supporting strategy use and primary and secondary outcomes will be extracted as reported in studies and may include weighted mean differences or standardized mean differences for continuous outcomes; odds ratios (ORs), relative risks (RRs), and risk differences for binary outcomes; and hazard ratios (HRs) for time-to-event data. Where studies do not provide effect estimates and their 95% CIs (or data required to calculate these measures) within the text or summary tables but summarize this information within figures, data will be extracted independently by two investigators using ImageJ software (National Institutes of Health) [72] and then their results will be averaged across investigators. Where data are not provided in the text, summary tables, or figures, these data will be sought from the supplementary materials of included trials or from trial registration repositories. In cases where data cannot be found using the above methods, study authors will be contacted in a final attempt to obtain all relevant study data. Discrepancies between investigators regarding data extraction will be resolved by consensus.

**Table 5:** Data items to be extracted from included studies when reported for systematic review examining implementation strategies for improving health care provider prescription of guideline-recommended cardiovascular medications to adults with atherosclerotic occlusive disease.

Data item theme	Items to be extracted
Study design	Country of origin Recruitment period Study design (eg, RCT^a^ or cluster-RCT) Setting (eg, primary, secondary, or tertiary) Number of health care providers, patients, or clusters Follow-up duration Clinical practice guidelines followed (eg, 2016 American College of Cardiology/American Heart Association PAD^b^ guidelines [7])
Prescriber characteristics	Health care provider specialty (cardiovascular specialist or noncardiovascular specialist) Health care provider subspecialty (eg, family medicine, general internal medicine, cardiology, or vascular surgery) Number of female health care providers Mean years of health care provider practice
Patient characteristics	Number of patients with different atherosclerotic occlusive diseases (cerebrovascular disease, coronary artery disease, PAD, or polyvascular disease) Mean or median patient age Mean or median patient BMI Number of female patients Number of non-White patients Number of patients with low socioeconomic status^c^ Number of past or current smokers Whether patients were inpatients, outpatients, or long-term care residents Number of patients who are overweight^d^ or obese^e^ Number of patients residing in rural areas Number of patients with specific comorbidities (dyslipidemia, diabetes mellitus, hypertension, congestive heart failure, chronic kidney disease, or chronic obstructive pulmonary disease)
Guideline-recommended medications	Classes of guideline-recommended medications examined (antiplatelets, statins, antihypertensives, or all medications) Types of guideline-recommended medications examined (eg, clopidogrel, simvastatin, or β-blockers)
Implementation strategies	Implementation strategy classification according to refined Effective Practice and Organization of Care taxonomy [61]
Effect measures	Effect measures (eg, weighted mean differences, standardized mean differences, odds ratios, relative risks, risk differences, or hazard ratios) and measures of variability (eg, 95% CIs) describing the effect of implementation strategies on primary and secondary outcomes
Risk of bias	Risk of bias for the 7 domains of the Cochrane risk of bias tool [71]

^a^RCT: randomized controlled trial.

^b^PAD: peripheral artery disease.

^c^As defined by study authors.

^d^Defined as a BMI ≥25 kg/m^2^.

^e^Defined as a BMI ≥30 kg/m^2^.

**Table 6:** Data items to be extracted from included studies when reported for systematic review examining adherence-supporting strategies for improving patient adherence to guideline-recommended cardiovascular medications in adults with atherosclerotic occlusive disease.

Data item theme	Items to be extracted
Study design	Country of origin Study design (eg, RCT^a^ or cluster-RCT) Setting (eg, primary, secondary, or tertiary) Recruitment period Number of patients, prescribers, or clusters Clinical practice guidelines followed (eg, 2022 Canadian Cardiovascular Society PAD^b^ guidelines [9]) Duration of follow-up
Patient characteristics	Atherosclerotic occlusive disease diagnoses (cerebrovascular disease, coronary artery disease, PAD, or polyvascular disease) Whether patients were inpatients, outpatients, or long-term care residents Mean or median age Mean or median BMI Number of patients with low socioeconomic status^c^ Number of female patients Number of non-White patients Number of past or current smokers Number of patients who are overweight^d^ or obese^e^ Number of patients residing in rural areas Number of patients with specific medical comorbidities (dyslipidemia, diabetes mellitus, hypertension, congestive heart failure, chronic kidney disease, or chronic obstructive pulmonary disease)
Prescriber characteristics	Specialty of health care providers (cardiovascular specialist or noncardiovascular specialist) Subspecialty of health care providers (eg, family medicine, general internal medicine, cardiology, or vascular surgery) Mean or median years of health care provider practice Number of female health care providers
Guideline-recommended medications	Classes of guideline-recommended medications examined (statins, antiplatelets, antihypertensives, or all guideline-recommended medications) Types of guideline-recommended medications examined (eg, aspirin, rosuvastatin, or angiotensin-converting enzyme inhibitors)
Adherence-supporting strategies	Adherence-supporting strategy classification according to the Behavior Change Wheel [62]
Effect measures	Effect measures (eg, weighted mean differences, standardized mean differences, odds ratios, relative risks, risk differences, or hazard ratios) and applicable variation measures (eg, 95% CIs) describing the effect of adherence-supporting strategies on primary and secondary outcomes
Risk of bias	Risk of bias for 7 domains of the Cochrane risk of bias tool [71]

^a^RCT: randomized controlled trial.

^b^PAD: peripheral artery disease.

^c^As defined by study authors.

^d^Defined as a BMI ≥25 kg/m^2^.

^e^Defined as a BMI ≥30 kg/m^2^.

### Risk of Bias

Two independent investigators will assess the risk of bias for all included trials using the Cochrane risk of bias tool [71]. Discrepancies between investigators regarding risk of bias assessment will be resolved by consensus.

### Data Synthesis

#### Qualitative

Before considering meta-analyses, we will first perform thematic clustering [73] of all included studies. We will classify implementation or adherence-supporting strategies used within both the intervention and comparator arms of identified trials based on (1) the levels and categories of the refined Effective Practice and Organization of Care (EPOC) taxonomy [61] (for implementation strategies for improving health care provider prescription); or (2) the nine intervention functions (education, persuasion, incentivization, coercion, training, restriction, environmental restructuring, modelling, and enablement) and seven policy categories (communication or marketing, guidelines, fiscal, regulation, legislation, environmental or social planning, and service provision) of the Behavior Change Wheel (BCW) [62] (for adherence-supporting strategies for improving patient adherence). The refined EPOC taxonomy was selected because it was designed to assist reviewers in selecting papers for inclusion in systematic reviews of implementation strategies [61]. It has also been used in systematic reviews examining RCTs of implementation strategies for patients with other chronic conditions [74,75] and allows for an adequate description of complex and multiple implementation strategies [61]. The BCW was selected because it was constructed by behavior change experts through systematic analysis and synthesis of 19 previously existing adherence-supporting strategy classification frameworks [62]. The BCW also satisfies three usefulness criteria not completely satisfied by any of the 19 individual frameworks on which it is built (comprehensiveness, coherence, and links to an overarching model of behavior) and can classify every adherence-supporting strategy that has been developed [62]. Further, linkage to an overarching model of behavior (the capability, opportunity, and motivation model) may allow for the selection of adherence-supporting strategies more likely to achieve the desired behavior change [62]. The BCW has also been shown to be reliable for classifying adherence-supporting strategies across a wide range of clinical contexts [62]. Implementation and adherence-supporting strategies will be classified by 2 independent investigators. Disagreements regarding implementation or adherence-supporting strategy classification will be resolved through consultation with a health psychologist.

Following implementation and adherence-supporting strategy classification, we will tabulate studies [73] based on the implementation strategies contained within their intervention and comparator arms. This will allow us to narratively summarize [73] how the implementation or adherence-supporting strategies used within the intervention and comparator arms of included studies differ regarding (1) the type and number of implementation or adherence-supporting strategies used; or (2) the intensity and duration of implementation or adherence-supporting strategy use. It will also allow us to describe which and how frequently specific implementation or adherence-supporting strategies are used in combination (ie, how frequently they co-occur together in complex implementation or adherence-supporting strategies). Studies will also be tabulated [73] by the classes of guideline-recommended cardiovascular medications that their implementation or adherence-supporting strategies are designed to improve the prescription of or adherence to, the predominant diagnoses of the patients included in this study (CVD, CAD, PAD, and polyvascular disease), and whether their implementation strategy was aimed at cardiovascular or noncardiovascular specialists (for implementation strategies for improving health care provider prescription).

#### Quantitative

Descriptive data will be summarized using weighted means and SDs, medians and IQRs, or counts and percentages where appropriate. Where minimal clinical interstudy heterogeneity exists [76], DerSimonian and Laird [77] random effects models will be used to pool effect estimates whenever two or more studies report on the effect of implementation or adherence-supporting strategies of the same type (according to the refined EPOC taxonomy [61] [underprescription systematic review] or the BCW [62] [nonadherence systematic review]) on similar outcomes. We will preferentially summarize the pooled effects of implementation or adherence-supporting strategies on the primary and secondary outcomes using RRs for dichotomous outcomes, weighted mean differences for continuous outcomes, and HRs for time-to-event outcomes (along with their 95% CIs). Where substantial interstudy clinical heterogeneity exists [76], we will summarize the results of included studies narratively [73] by reporting effect estimates (along with their 95% CIs) describing the effectiveness of implementation or adherence-supporting strategies at improving primary or secondary outcomes.

Where studies report effect measures other than RRs (ie, ORs or HRs), we will use corresponding raw data describing the cumulative incidence of underprescription or nonadherence or secondary outcomes to calculate RRs from ORs or HRs using validated methods [78,79] as has been done previously [80,81].

For included cluster-RCTs, we will calculate a design effect (DE) for each study using an established formula (*DE = 1+ [n − 1] × ρ*) [82,83]. This DE will then be used to reduce the total sample size to an “effective sample size” (*effective sample size = n/DE*) to account for clustering effects [82]. If an interclass correlation coefficient (ICC; ρ) is not reported in one or more of the included studies, a single conservative ICC value will be used [84]. The value of this single conservative ICC will be based on ICCs from similar studies [84] and will be imputed separately for RCTs examining provider-level or health care system-level strategies (ie, those targeting underprescription) and patient-level strategies (ie, those targeting nonadherence) [85].

Interstudy heterogeneity will be assessed by inspecting forest plots and calculating *I*² statistics [86]. We will consider *I*² statistics >25%, >50%, and >75% to represent low, moderate, and high degrees of heterogeneity, respectively [86]. We will also conduct stratified meta-analyses and meta-regressions using several prespecified risk factors for underprescription and nonadherence selected based on the findings of related systematic reviews [87-92] and methodological considerations [15,16,93-103]. The following risk factors will be used within these analyses to determine whether they may be effect modifiers: (1) whether the predominant atherosclerotic occlusive disease was CAD or another atherosclerotic occlusive disease (CVD, PAD, or polyvascular disease) [15,16]; (2) whether most prescribers were cardiovascular specialists (eg, cardiologists or vascular surgeons) or noncardiovascular specialists (eg, family physicians, general internal medicine physicians, pharmacists, or nurse practitioners) [96-98]; (3) whether studies originated from high or low-middle income countries [99,100] (according to the list provided by the World Bank in 2023) [93]; (4) the proportion of female [101-103], non-White [101-103], and low socioeconomic status [101-103] patients; (5) whether the mean age of patients was ≥65 years of age instead of <65 years of age (or whether ≥50% of the population was aged ≥65 years versus <50% of the population was aged ≥65 years) [101-103]; (6) study setting (primary care versus tertiary care) [101-103]; (7) whether participants were predominantly inpatients, outpatients, or long-term care residents (nonadherence systematic review only) [101,102]; (8) whether there was a high or unclear versus lower risk of bias related to random sequence generation, allocation concealment, and blinding of outcome assessors [73]; and (9) whether information regarding medication adherence was obtained using objective methods (eg, biochemical assays [94,95], electronic pill bottle openings [94,95]) or participant self-report [94] (eg, Morisky Medication Adherence Scale [104], Medication Adherence Report Scale [105] [nonadherence systematic review only]).

### Publication Bias and Small-Study Effects

The presence of publication bias will be evaluated using the Egger test and by visually inspected produced funnel plots from primary outcomes [106].

### Statistical Software

All analyses will be conducted using Stata Standard Edition (version 17.0; Stata Corp). Risk-of-bias graphics will be generated using Review Manager Systematic Review Software (version 5.4; The Cochrane Collaboration).

### Certainty in the Cumulative Evidence

We will use the GRADE (Grading of Recommendations, Assessment, Development, and Evaluation) [107] to rate certainty in outcome estimates. First, we will assess the risk of bias, imprecision, inconsistency, indirectness, and publication bias in outcome estimates [107]. The overall certainty in these estimates will then be judged as high (“further research is very unlikely to change our certainty in the estimate”), moderate (“further research is likely to have an important impact on our certainty in the estimate and may change the estimate”), or low (“further research is very likely to have an important impact on our certainty in the estimate and is likely to change the estimate”) [107].

### Ethical Considerations

These systematic reviews will examine previously published data and are therefore exempt from ethics approval at our institution.

## Results

**Figure 1:**
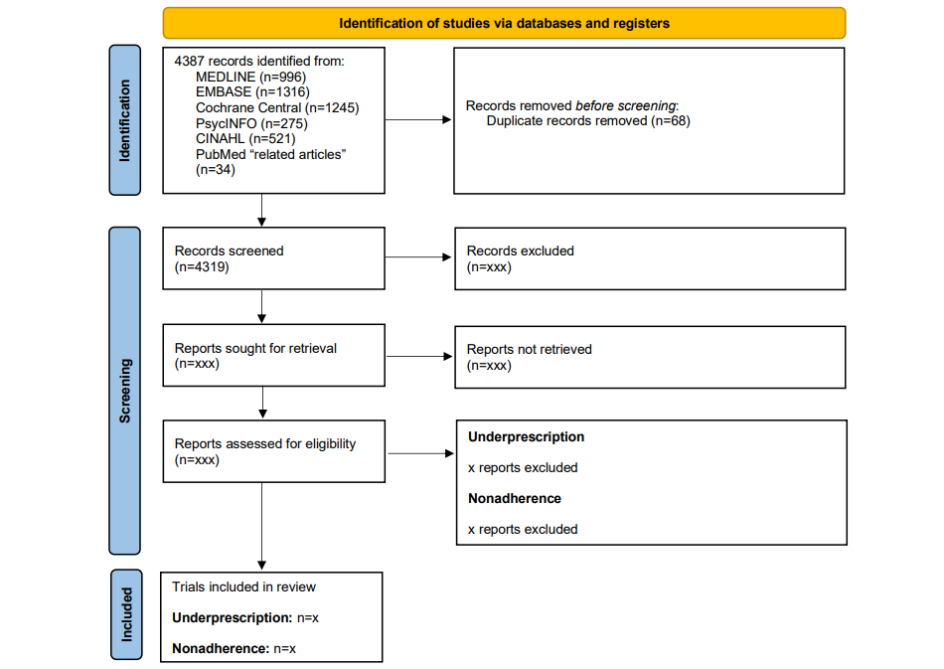
PRISMA flow diagram describing the flow of articles through the systematic reviews. PRISMA: Preferred Reporting Items for Systematic Reviews and Meta-Analyses.

We conducted the database and PubMed “related articles” search on June 6, 2023. Database searches identified 4353 total citations (n=996 from MEDLINE; n=1316 from Embase; n=1245 from Cochrane Central; n=275 from PsycINFO; and n=521 from CINAHL). The PubMed “related articles” feature identified another 34 citations. After removing duplicates, 4319 unique citations remain for title/abstract screening (see Figure 1 for our PRISMA [Preferred Reporting Items for Systematic Review and Meta-Analysis] flow diagram). We are currently in the process of performing title/abstract screening. We hope to complete analyses for the two systematic reviews by mid-2025.

## Discussion

### Future Directions and Anticipated Principal Results

Our systematic reviews will assess all RCTs examining the effectiveness of implementation and adherence-supporting strategies for improving health care provider prescription of, and patient adherence to, guideline-recommended cardiovascular medications among adults with atherosclerotic occlusive diseases. We also aim to determine the effectiveness of implementation strategies at improving medication prescription across different classes of health care providers working across different clinical practice settings. We anticipate that clinician education may be a particularly effective strategy for improving prescription among those working in primary care settings, given that these types of practitioners may have relatively reduced awareness of guideline recommendations for the management of patients with atherosclerotic occlusive disease relative to cardiovascular specialists [108]. As patients treated at tertiary medical centers tend to have complex electronic medical records [109], we anticipate that a facilitated relay of information to clinicians may be particularly effective for improving medication prescription among cardiovascular specialists and clinicians working in tertiary academic centers. Strategies anticipated to be promising for improving guideline-recommended cardiovascular medication prescription across different health care provider types and clinical practice settings include audit and feedback, provider reminder systems, and organizational changes given the promise these interventions have demonstrated for improving medication prescription among patients with varied cardiovascular diseases [87,88].

We will also examine whether the effectiveness of adherence-supporting strategies varies across patients with different types of atherosclerotic occlusive diseases (CVD, CAD, PAD, and polyvascular disease). In particular, identifying promising adherence-supporting strategies for improving medication adherence among patients with PAD is of crucial importance given that research has shown that those with PAD are less likely to be prescribed guideline-recommended medications compared to those with CVD or CAD [19]. As patients with PAD tend to have worse awareness of their condition and relevant secondary medical prevention options compared to those with other atherosclerotic occlusive diseases [110], we anticipate that patient education may be a particularly promising strategy for improving medication adherence among patients with PAD. Adherence-supporting strategies involving fiscal components (eg, medication copayment) may also be a particularly promising strategy for improving medication adherence among patients with PAD given the high costs of guideline-recommended cardiovascular medications [111]. Further, as patients with atherosclerotic occlusive disease tend to be older [53-55], strategies that remind patients to take their prescribed medications may serve as promising adherence-supporting strategies across patients with atherosclerotic occlusive disease in general.

### Comparison With Previous Work

Although systematic reviews have examined the effectiveness of implementation and adherence-supporting strategies for improving health care provider prescription of [87,88], and patient adherence to [89-92], guideline-recommended cardiovascular medications for atherosclerotic occlusive diseases, they have important limitations. One systematic review of implementation strategies for improving health care provider prescription [87] characterized its implementation strategies according to the EPOC taxonomy [112], which was developed to help authors classify implementation strategies in systematic reviews. However, it aggregated implementation strategies together to assess whether they collectively increased health care provider prescription of guideline-recommended medications instead of examining whether strategy effectiveness varied within different EPOC taxonomy [112] levels (ie, professional, organizational, financial, and regulatory). This systematic review [87] also only examined patients with CAD. Although a second systematic review [88] on the effectiveness of implementation strategies for improving health care provider prescription also characterized implementation strategies using the EPOC taxonomy [112], it examined the effectiveness of improving physician adherence to “guideline-recommended care” (eg, use of cardiovascular risk assessment tools or following guidelines for cardiovascular disease screening) in addition to medication prescription [88]. It also included patients with nonatherosclerotic cardiovascular diseases. Limitations of the existing systematic reviews of adherence-supporting strategies for improving patient adherence is that many include patients “at risk” of developing an atherosclerotic occlusive disease or with nonatherosclerotic cardiovascular diseases (eg, hypertension or congestive heart failure) [91,92]. Further, many only examine specific adherence-supporting strategies (eg, polypills, text-message reminders, or patient education) [89,90,92] or adherence to specific cardiovascular medications among patients with CVD or CAD [89-92]. Many also did not categorize their adherence-supporting strategies according to an established taxonomy [91]. Finally, many relevant RCTs have been completed since the publication of prior underprescription [87,88] and nonadherence [89-92] systematic reviews.

### Strengths and Limitations

Our proposed systematic review has potential limitations. First, we anticipate that it may be difficult to pool the results of some studies due to interstudy heterogeneity [113]. We anticipate that many relevant RCTs [21-48] may have reported different primary and secondary outcomes at different time points and using different outcome measurement scales. Should we be unable to pool our quantitative results, we will summarize our results narratively instead of using the methods outlined above [73]. Second, it may be argued that including patients with all forms of atherosclerotic occlusive disease in our study population may introduce clinical heterogeneity due to small differences in underlying disease pathophysiology [50-52] and epidemiology [53-55]. Nevertheless, the patient populations in our proposed systematic reviews are more similar regarding disease pathology, epidemiology, and medication requirements than others in previously published systematic reviews [87-92]. Further, these conditions commonly coexist within patients [56,57] and similar prescribers are involved in their medical management [58-60]. Finally, although the use of self-reported data to quantify medication adherence introduces the possibility of outcome misclassification bias [94], we plan on performing stratified meta-analysis and meta-regression to determine whether observed differences in medication adherence vary by whether studies quantified medication adherence using objective methods versus participant self-report.

### Conclusions

In conclusion, we will perform systematic reviews and meta-analyses of RCTs examining implementation and adherence-supporting strategies designed to improve health care provider prescription of, and patient adherence to, guideline-recommended cardiovascular medications among patients with atherosclerotic occlusive diseases. These systematic reviews will also determine the effect of these interventions on patient-important outcomes and health care resource use. Finally, they will determine whether effectiveness varies by patient characteristics, prescriber traits, and clinical practice setting.

## Appendix

Multimedia Appendix 1 PRISMA-*P* 2015 checklist. PRISMA-P: Preferred Reporting Items for Systematic Reviews and Meta-Analysis Protocols.

Multimedia Appendix 2 Database search strategies (June 6, 2023). MeSH: Medical Subject Heading.

## Abbreviations

AACTT: action, actor, context, target, time
BCW: Behavior Change Wheel
CAD: coronary artery disease
CVD: cerebrovascular disease
DE: design effect
EPOC: Effective Practice and Organization of Care
GRADE: Grading of Recommendations, Assessment, Development, and Evaluation
HR: hazard ratio
ICC: interclass correlation coefficient
MACE: major adverse cardiac event
MALE: major adverse limb event
OR: odds ratio
PAD: peripheral artery disease
PRISMA: Preferred Reporting Items for Systematic Review and Meta-Analysis
PRISMA-P: Preferred Reporting Items for Systematic Review and Meta-Analysis Protocols
RCT: randomized controlled trial
RR: relative risk

## Data Availability

Data from the systematic review will be provided by study authors upon reasonable request.
