# Supplementary material for: Strategies to Improve Health Care Provider Prescription of and Patient Adherence to Guideline-Recommended Cardiovascular Medications for Atherosclerotic Occlusive Disease: Protocol for Two Systematic Reviews and Meta-Analyses of Randomized Controlled Trials
Source: JMIR Res Protoc. 2025 Jan 16;14:e60326. doi: 10.2196/60326 (PMC11783033; doi:10.2196/60326)
Supplement: Multimedia Appendix 2 [file resprot_v14i1e60326_app2.docx]

| **Search Theme** | **Search Terms** | | | | | |
| --- | --- | --- | --- | --- | --- | --- |
|  | **Ovid MEDLINE, Cochrane Central,**  **Evidence-Based Medicine Reviews and APA PsycInfo** | | **Ovid EMBASE** | | **CINAHL Complete** | |
|  | **Exploded MeSH^a^ Terms** | **Title and Subject Keywords** | **Exploded Emtree Terms** | **Title and Subject Keywords** | **Query** | **Limiters/Expanders** |
| Atherosclerotic occlusive disease | Peripheral arterial disease OR peripheral vascular diseases OR coronary artery disease OR coronary disease OR cardiovascular diseases OR cerebrovascular disorders OR carotid artery diseases OR arterial occlusive diseases OR occlusive disease | ((arterioscleros* or coronary artery disease* or atheroscleros* or coronary heart disease*)) ((cerebrovascular disease* or cardiovascular disease*)) ((limb adj2 isch?em*)) ((periph* adj2 arter* adj2 disease*)) ((arter* occlusive disease* or arter* obstructive disease*)) ((peripher* vascular disease* or intermittent claud*)) ((isch?em* heart disease* or stable angina)) ((stroke or brain isch?em* or carotid stenos* or carotid arter* disease*)) | Peripheral occlusive artery disease OR peripheral vascular disease OR coronary artery disease OR cardiovascular disease OR cerebrovascular disease OR cerebrovascular accident OR carotid artery disease OR carotid artery obstruction OR arterial occlusive disease OR arterial obstructive disease | ((arterioscleros* or coronary artery disease* or atheroscleros* or coronary heart disease*)) ((cerebrovascular disease* or cardiovascular disease*)) ((limb adj2 isch?em*)) ((periph* adj2 arter* adj2 disease*)) ((arter* occlusive disease* or arter* obstructive disease*)) ((peripher* vascular disease* or intermittent claud*)) ((isch?em* heart disease* or stable angina)) ((stroke or brain isch?em* or carotid stenos* or carotid arter* disease*)) | Peripheral vascular diseases OR coronary arteriosclerosis OR coronary disease OR cardiovascular diseases OR cerebrovascular disorders OR carotid artery diseases OR stroke, OR arterial occlusive diseases | ((arterioscleros* or coronary artery disease* or atheroscleros* or coronary heart disease*)) ((cerebrovascular disease* or cardiovascular disease*)) ((limb N2 ischem*)) ((periph* N2 arter* N2 disease*)) ((arter* occlusive disease* or arter* obstructive disease*)) ((peripher* vascular disease* or intermittent claud*)) ((ischem* heart disease* or stable angina)) ((stroke or brain isch?em* or carotid stenos* or carotid arter* disease*)) |
| Guideline-recommended medication prescription or adherence | Guideline adherence OR medication adherence OR treatment adherence and compliance OR drug prescriptions OR evidence-based medicine | ((therap* or management or therapeutic* or medication* or prescription* or prescrib* or drug)) ((treatment or prescribing or therapy or medication) adj5 adheren*)) ((adheren* adj5 intervent*)) ((treatment or prescribing or therapy or medication) adj3 complian*)) ((evidence base* adj2 (therap* or treatment* or prescrib* or prescription*)) ((implement*or quality improvement/ or quality improvement)) ((improv* adj4 (prescrib* or prescription or aherence)) | Protocol compliance OR medication compliance OR prescription OR evidence based medicine OR total quality management | ((therap* or management or therapeutic* or medication* or prescription* or prescrib* or drug)) ((treatment or prescribing or therapy or medication or prescription) adj5 adheren*) or adheren*)) ((adheren* adj5 intervent*)) ((treatment or prescribing or therapy or medication or prescription) adj3 complian*) or complian*) ((evidence base* adj2 (therap* or treatment* or prescrib* or prescription*)) ((implement* or quality improvement)) ((improv* adj4 (prescrib* or prescription or aherence)) | Guideline adherence OR medication compliance OR prescriptions, drug OR medical practice, evidence-based OR nursing practice, evidence-based OR implement OR quality improvement OR | (therap* or management or therapeutic* or medication* or prescription* or prescrib* or drug)) ((treatment or prescribing or therapy or medication) N5 adheren*)) ((treatment or prescribing or therapy or medication) N3 complian*)) ((adheren* N5 intervent*)) ((evidence base* N2 (therap* or treatment* or prescrib* or prescription)) ((implement*)) ((quality improvement)) ((improv* N4 (prescrib* or prescription or aherence)) |
| Randomized controlled trial | Randomized controlled trial OR controlled clinical trial | ((random* or placebo)) | Random OR placebo OR double-blind | ((random* or placebo* or double-blind*)) | Randomized Controlled Trials OR Double-Blind Studies OR Therapeutic Trials OR Triple-Blind Studies OR Random OR Placebo OR Random Assignment OR Control Group OR Trial OR Intervention Trials | ((random*)) ((placebo)) ((control* N2 (stud* or group* or trial*)) |

^a^Where MeSH, Medical Subject Heading.
